# Desmosomes in Developing Human Epidermis

**DOI:** 10.1155/2010/698761

**Published:** 2010-06-01

**Authors:** Sirkku Peltonen, Laura Raiko, Juha Peltonen

**Affiliations:** ^1^Department of Dermatology, University of Turku and Turku University Hospital, PL 52, 20521 Turku, Finland; ^2^Department of Cell Biology and Anatomy, University of Turku, 20520 Turku, Finland

## Abstract

Desmosomes play important roles in the cell differentiation and morphogenesis of tissues. Studies on animal models have greatly increased our knowledge on epidermal development while reports on human developing skin are rare due to the difficult accessibility to the samples. Although the morphology of periderm cells and the process how the epidermis develops very much resemble each other, the timetable and the final outcome of a mature human epidermis markedly differ from those of murine skin. Even the genetic basis of the junctional components may have profound differences between the species, which might affect the implementation of the data from animal models in human studies. The aim of this review is to focus on the development of human skin with special emphasis on desmosomes. Desmosomal development is mirrored in perspective with other simultaneous events, such as maturation of adherens, tight and gap junctions, and the basement membrane zone.

## 1. Introduction

The literature on developing human epidermis is limited, collectively not exceeding 100 cases in the reports covering the fetal age. The development of human skin has been studied at the morphological level in quite detail by electron microscopy [1–3]. The timetable for the formation of epidermal architecture is based on the evaluation of sixty human fetuses, age 7–20 weeks [2]. Sparsely located desmosomes are detected already in the samples from the youngest fetuses, and during the maturation the density of desmosomes increases [2, 4].

Since desmosomes are relatively easily identifiable by their ultrastructural appearance, they were the first specific cell junctions recognized in human skin by electron microscopy. The other cell junctions, adherens, tight, and gap junctions, were originally identified and named in simple epithelia using electron microscopy, but their ultrastructural characteristics in simple epithelia are not directly applicable to those of stratified epithelia, such as epidermis. The recognition of a number of biomarkers of cell junctions and subsequent availability of specific antibodies at 80's and 90's enabled investigation of the junctional proteins of epidermis using immunohistochemical approaches at light and electron microscopic levels. Immunolocalization studies thus helped the identification of desmosomal, adherens junction, gap junction and tight junction, components in developing epidermis. Although some studies regarding regulation of development of human skin have been published, very little is known about the regulatory signals regarding developmental regulation of human skin.

## 2. Morphological Development of Human Epidermis

**Figure 1:**
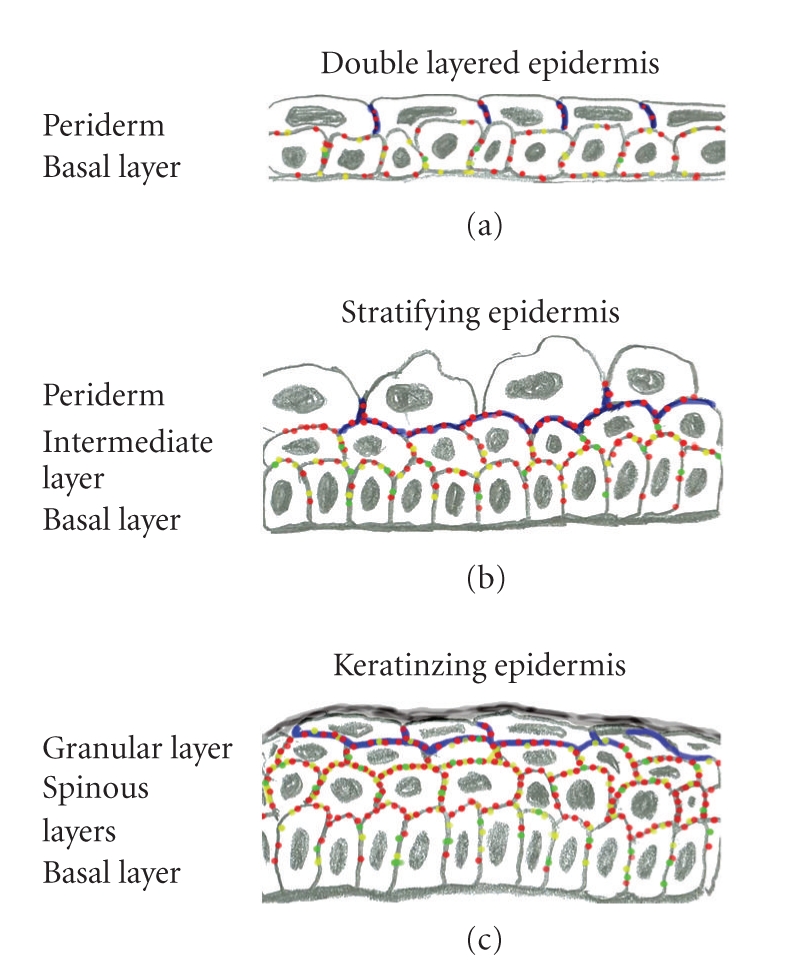
Schematic representation of developing human epidermis. (a) Two cell layers, peridermal cells and basal cells at the first trimester (8 weeks). (b) Three cell layers at 11 weeks. The periderm cells become elevated. (c) By 21–24 weeks all the cell layers of mature epidermis are present. Tight junctions are shown in blue, desmosomes in red, adherens junctions in yellow, and green indicates gap junctions. The density of junctions increases during the maturation.

Human epidermis is derived from a single layer of embryonic surface ectoderm. The ectoderm proliferates in the 4th week of development and produces two layers of cells [1, 2] (Figure 1). The inner layer of cells is the basal layer while the outer layer is called the periderm, and proliferation takes place in both cell layers [2]. In the 11th week of EGA, the basal layer produces a new intermediate cell layer between itself and the periderm which marks the beginning of stratification and more complicated differentiation of the epidermis. The periderm cells in contrast, cease dividing in the first trimester, become larger and elevated, and exhibit rounded blebs on their outer surfaces [2]. The periderm cells form a cornified cell envelope in the three-layered stage of development [5, 6]. By 21–24 weeks EGA, the intermediate cell layer has apparently given rise to the definitive three layers of the outer epidermis: the spinous, the granular, and the cornified cell layers. As the keratinization proceeds, the periderm is gradually shed into the amniotic fluid by the beginning of the last trimester [3]. The periderm cells display characteristics consistent with apoptosis prior to being sloughed off [7]. Cornified cell envelope is formed in the upper cell layers of epidermis after the shedding of the periderm cells [5, 6]. Mice and man show clear differences in the development of the epidermis including the time schedule, maturity at newborn and differences at the genetic level. For example, mice posses three dsg1 genes with distinct epidermal expression patterns whereas there is only a single human DSG1 gene.

## 3. Formation of Intercellular Junctions in Early Two-Layered Epidermis: 4–9 Weeks

Desmosomes are easily detectable in transmission electron microscopy. Ultrastructural studies have revealed desmosomes at 5 weeks of EGA between the basal and periderm cells [2]. It is possible that desmosomes exist earlier but this has not been verified because of lack of samples representing earlier time points. Formation of desmosomes is thus a very early event well preceding, for example, the maturation of the basement membrane zone. In the youngest fetal samples investigated, the desmosomes are widely separated [2] and evaluation of the electron microscopic images suggests that the desmosomal plaque is considerably thinner and less prominent than in later developmental stages, although this was not highlighted in the original publication.

**Figure 2:**
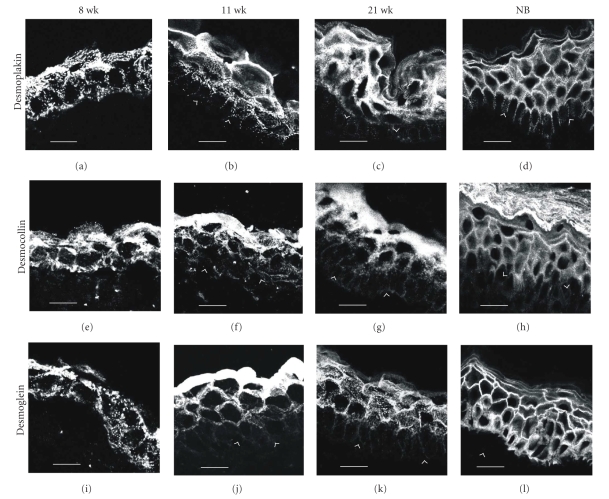
Expression and localization of desmosomal proteins in developing human skin at 8, 11, and 21 weeks of EGA and at newborn age (NB). Note the gradual increase in the density of desmosomes. At 8 weeks EGA, the epidermis is composed of basal and peridermal cell layers (a, e, i). Antibodies to desmoplakin (a), desmocollin (e), and desmoglein (i) label cell membranes of basal and peridermal cells. Note also immunolabeling in the dermal-epidermal junction. At 11 weeks EGA, the intermediate cell layer has developed in the epidermis (b, f, j). An intense signal for desmoplakin (b), pan-desmocollin (f), and pan-desmoglein (j) is apparent in the peridermal cells. Intermediate cells also show desmosomal antigens, while lateral membranes of basal cells are almost devoid of these desmosomal proteins. At 21 weeks EGA, the peridermal cell layer has been shed and the epidermis is composed of the four definitive layers of epidermis (c, g, k). Desmoplakin (c), desmocollin (g), and desmoglein (k) antibodies label all the cell layers, the basal cells being only weakly labelled (*arrowheads point to the *dermal-epidermal junction; *bars *(a, e, i) 10 *μ*m, (b, c, d, f, g, h, j, k, i) 20 *μ*m).

The protein composition of early fetal desmosomes has been studied at 5 weeks using serum from pemphigus patients, but no intercellular fluorescence was detected at that time [8]. Correspondingly, pemphigus sera revealed positive immunoreaction only after 11 weeks in the study by Lane et al. [9]. Thus, the presence of desmogleins could not be proved in the samples of earliest developmental points studied. However, at 8 weeks indirect immunofluorescence with antibodies to desmoplakin, pan-desmocollin, and pan-desmoglein showed punctate labeling associated with plasma membranes of peridermal and basal cells [4] (Figures 1 and 2), indicating that developing desmosomes have the elements for transmembrane and plaque parts. The suggestive intermediate filaments binding to the desmosomal plaques in basal cells are CK5 and CK14 which can be first detected between 8 and 10 weeks [10] while the periderm cells contain CK19 and CK8 [11]. 

At eight weeks of EGA, desmosomal proteins were also localized to the basal plasma membrane of the basal cells suggesting that separation of cell membranes to basal and apicolateral compartments had not taken place at this time. At this time, immunolabeling for *β*4 integrin shows widely distributed spots [4], instead of a linear labeling of mature basement membrane zone. Structural hemidesmosomes are also not seen in the electron microscopy [2, 12, 13]. These findings support the view that the polarity of the basal cells has not developed yet. It is however known that at 5 weeks EGA, the basement membrane zone is composed of a basal cell plasma membrane, lamina lucida, and lamina densa [12] which contain laminin and type IV collagen [9, 14, 15]. *β*1 integrin can also be seen in the periphery of the basal cells, including the basal and apicolateral plasma membranes [4, 16, 17]. 

In vitro studies on human primary keratinocytes have shown that adherens junctions precede the development of desmosomes [18]. Classical cadherins are important in the initiation of intercellular junction formation, and regulation of desmosome *assembly depends at least to some extent, on expression of classical cadherins* [19], Tinkle et al. [20]. In two-layered developing human epidermis of eight weeks, E-cadherin is expressed in the periphery of basal cells, including the basal aspect, and in the periphery of peridermal cells. E-cadherin and P-cadherin are also present in the intercellular junctions of the basal and peridermal cells [4, 10, 21]. The same localization was also noted for *α* catenin, vinculin, and *α* actinin [4]. This indicates that the prerequisite for desmosome formation in the form of adherens junction components is available and thus the formation of desmosomes may follow the same sequence of events as has been described in vitro. It should be noted that the adherens junctions were not *reported* in the ultrastuctural studies. This is, however, not surprising since the plaque of the adherens junctions is much less prominent than the desmosomal plaque and is difficult to visualize even in the adult epidermis [22]. 

It should also be noted that tight junctions are visible between the neighboring peridermal cells [23]. Of the components of tight junctions, ZO-1 and occludin have been demonstrated in the cell junction complexes of peridermal cells between 8 and 21 weeks of EGA [23]. Tight junctions are responsible for the epidermal diffusion barrier at this age when mature stratum corneum does not exist. Providing diffusion barrier for the epidermis might also be an important basic function of the periderm. 

Gap junctions, composed of connexin (Cx) subunits, are channels that allow intercellular communication between adjacent cells. They are considered to play a key role in the regulation of cell proliferation and differentiation. The major Cx subtypes in human skin are Cx26 and Cx43 [24]. The first connexin type expressed already at 7 weeks is Cx26 [25] and sparse gap junctions containing connexin 43 are present at eight weeks [4]. Formation of gap junctions increases while the epidermis develops and matures, suggesting that gap junctions may play an important role in fetal skin development.

## 4. Initiation of Stratification and Differentiation of the Basal Cells between 9 and 20 Weeks

Between 9 and 20 weeks, the intermediate filament bundles associated with desmosomes become larger and more prominent, and the number of desmosomes increases [2, 11]. The new intermediate layer of cells contains more desmosomes and more prominent keratin filaments than the basal and periderm cells. The putative binding partners for desmosomal plaque proteins are CK5 and CK14 in the basal cells, while the intermediate cells change the expression to CK1 and CK10 [11]. As the stratification proceeds, CK1 and CK10 are expressed in all suprabasal cell layers. CK8 and CK19 are still expressed in both the basal and periderm cell layers at this stage of development, but disappear with keratinization, by about 24 weeks EGA [11]. 

When the stratification takes place, labeling of the basal cells for desmoplakin, pan-desmocollin, and pan-desmoglein becomes very weak and only some distinct spots of desmosomes are visible between the basal cells [4] (Figures 1 and 2). The basal cells have acquired their polarity and the desmosomal proteins have disappeared from the dermal-epidermal junction. Both of these characteristics resemble those of mature epidermis. The intermediate cells express all the desmosomal proteins studied which is in accordance with the presence of numerous desmosomes in the mature spinous cell layers. A study using pemphigus sera suggests that at this developmental state desmoglein3 is present in the basal and intermediate layers [9] while the peridermal cells merely show diffuse cytoplasmic labeling for many proteins studied [4, 9]. By 21 weeks, EGA the labeling pattern for desmosome components becomes more continuous which indicates the presence of more numerous desmosomes at the cell-cell contacts (Figure 2). 

During stratification the expression profiles of adherens junction proteins undergoes minor changes. The basal cells continue to express both E- and P-cadherins, while the intermediate cells express only E-cadherin [21]. The *α*-actinin disappears from the peridermal cells already by 11 weeks, while being prominently expressed in the junctions connecting basal and intermediate cells throughout the development [4]. *α*-catenin and vinculin are expressed in all the three epidermal layers. Between 13 and 21 weeks, as the number of intermediate cell layers increases, the expression pattern remains essentially the same and by 21 weeks EGA, the labeling patterns of adherens junction antigens resemble that of neonatal epidermis. The expression of *β*1 integrin continues in the basal cell layer as described earlier [16, 26].

Simultaneously with the initiation of stratification, the basement membrane zone goes through major changes as hemidesmosomes and anchoring fibrils begin to shape [12, 13]. Between 9 and 15 weeks (EGA), the number of hemidesmosomes is increased by about fourfold, they are matured and become increasingly associated with intermediate and anchoring filaments [9, 14]. By 20 weeks (EGA), the expression of *α*6*β*4 integrin becomes mostly concentrated at the basal surface of the basal cells [4, 26, 27]. The basement membrane becomes continuous and thicker.

## 5. Keratinization of the Epidermis and Shedding of the Periderm Cells after 20 Weeks of EGA

By approximately 21–24 weeks EGA, the intermediate cell layer has proliferated and produced the definitive three layers of the outer epidermis: the spinous, the granular, and the cornified cell layers [28] (Figure 1). As the keratinization proceeds, the periderm is gradually shed into the amniotic fluid [3]. After 20 gestational weeks the morphology of the epidermis increasingly resembles that of a newborn. The expression patterns of the cell junction and basement membrane components remain essentially the same and little alterations have been noted during this period of epidermal development. Desmosomes become more densely located in the spinous cell and granular cell layers. This is also shown in the immunofluorescence labeling for desmosomal antibodies which gradually reveal more continuous pattern in the cell-cell contacts [4] (Figures 1 and 2). The uppermost granular cell layer and the lowest layer of the stratum corneum, as well as the lateral plasma membranes of the granular layer are interconnected with tight junctions that are *intermingled* with numerous desmosomes [23, 29]. The density of gap junctions increases [4].

## 6. Regulation of the Development of Human Epidermis

Even though some of the signaling molecules and pathways are universally conserved, marked differences between human and mouse exist. Thus, findings in mice are not directly applicable for human development, or diseases. Yet, only few reports concerning the regulation of the differentiation of fetal human skin are available, and selected ones of those are reviewed here.

Wnt/*β* catenin signaling is known to play important roles in the development of skin and its appendages [30]. One study which was based on five fetal skin samples aged over 20 weeks, showed expression of Wnt3a, active *β* catenin and Dkk1 in fetal epidermis [31]. The authors suggest that Wnt/*β* catenin, signaling thus plays a role in human fetal skin development and homeostasis. Further studies would however be needed in order to investigate this pathway in more detail and at earlier time points. 

Desmosome assembly and disassembly are regulated, for example, by calcium and cross-talk with adherens junctions (for review see [32]). Adherens junctions and tight junctions are also regulated by calcium [33]. The effect of calcium is at least in part mediated by the epidermal calcium gradient which results in typical calcium concentrations in different epidermal cell layers [34]. However, no evidence on the epidermal calcium levels in fetal skin is available. 

The epidermal growth factor (EGF) family comprises multiple mediators such as transforming growth factor *α*, amphiregulin, heparin binding-EGF, and epiregulin, which are crucially involved in the tissue-specific proliferation/differentiation homeostasis [35]. TGF*α* is believed to play a role in cell proliferation and differentiation via an autocrine mechanism. It exerts its effects on cells through binding to the epidermal growth factor receptor (EGFR) [35]. TGF*α* has showed a vertical progressive increase in expression in the fetal skin of 14, 20, and 34 weeks [36]. In contrast to normal adult human skin in which the EGFR is primarily restricted to the basal and immediately suprabasal keratinocytes, the fetal epidermis showed a persistent expression of EGFR in all cell layers [37]. Based on these observations it has been suggested that TGF*α* and EGFR interact strictly to promote skin development during fetal period. 

Periderm is an embryonic- and fetal-specific transient cell layer which is destined to detach into the amniotic fluid. During human skin development periderm cells and incompletely keratinized cells are replaced by differentiating keratinocytes. The fate of the peridermal cells has been shown to take place via apoptosis [7]. Immunohistochemical localization of transglutaminases in fetal periderm and intermediate epidermal cells coincides with DNA fragmentation indicating that apoptosis is involved in deletion of these stage-specific cells. The detachment of periderm cells also has to involve disassembly of the desmosomes.

## 7. Conclusions

Studies on human skin are needed to relate the findings of animal studies with human development, physiology, and pathological conditions. Detailed timetables of the expression of several cell junction components are available, and based on these studies it is likely that the development of desmosomes is synchronized with the maturation of other junction types. However, studies on even the most profound mechanisms of differentiation of human skin are still lacking.

## Abbreviations

EGA: Estimated gestational age = the time from fertilization
EGF: Epidermal growth factor
TNF: tumor necrosis factor.
